# A Method for Sporulating Budding Yeast Cells That Allows for Unbiased Identification of Kinase Substrates Using Stable Isotope Labeling by Amino Acids in Cell Culture

**DOI:** 10.1534/g3.114.013888

**Published:** 2014-08-27

**Authors:** Ray Suhandynata, Jason Liang, Claudio. P. Albuquerque, Huilin Zhou, Nancy M. Hollingsworth

**Affiliations:** * Department of Biochemistry and Cell Biology, Stony Brook University, Stony Brook, New York 11794-5215; † Ludwig Institute for Cancer Research, University of California, San Diego, La Jolla, California 92093; ‡ Department of Chemistry and Biochemistry, University of California, San Diego, La Jolla, California 92093; § Department of Cellular and Molecular Medicine, University of California, San Diego, La Jolla, California 92093

**Keywords:** phosphorylation, Mek1 kinase, meiosis, mass spectrometry, SILAC

## Abstract

Quantitative proteomics has been widely used to elucidate many cellular processes. In particular, stable isotope labeling by amino acids in cell culture (SILAC) has been instrumental in improving the quality of data generated from quantitative high-throughput proteomic studies. SILAC uses the cell’s natural metabolic pathways to label proteins with isotopically heavy amino acids. Incorporation of these heavy amino acids effectively labels a cell’s proteome, allowing the comparison of cell cultures treated under different conditions. SILAC has been successfully applied to a variety of model organisms including yeast, fruit flies, plants, and mice to look for kinase substrates as well as protein–protein interactions. In budding yeast, several kinases are known to play critical roles in different aspects of meiosis. Therefore, the use of SILAC to identify potential kinase substrates would be helpful in the understanding the specific mechanisms by which these kinases act. Previously, it has not been possible to use SILAC to quantitatively study the phosphoproteome of meiotic *Saccharomyces cerevisiae* cells, because yeast cells sporulate inefficiently after pregrowth in standard synthetic medium. In this study we report the development of a synthetic, SILAC-compatible, pre-sporulation medium (RPS) that allows for efficient sporulation of *S. cerevisiae* SK1 diploids. Pre-growth in RPS supplemented with heavy amino acids efficiently labels the proteome, after which cells proceed relatively synchronously through meiosis, producing highly viable spores. As proof of principle, SILAC experiments were able to identify known targets of the meiosis-specific kinase Mek1.

Protein phosphorylation is critical for many different aspects of meiotic chromosome behavior in *Saccharomyces cerevisiae*. Recent studies have shown that conserved kinases such as cyclin-dependent kinase 1, Cdc7-Dbf4, casein kinase 1 (Hrr25), and the polo-like kinase, Cdc5, are involved in a variety of meiosis-specific processes, including the initiation of meiotic recombination, resolution of recombination intermediates, synaptonemal complex disassembly, mono-orientation of homologous pairs of sister chromatids at Meiosis I, and/or cleavage of cohesion at the onset of Anaphase I (Henderson *et al.* 2006; Lo *et al.* 2008; Matos *et al.* 2008; Sourirajan and Lichten 2008; Wan *et al.* 2008; Katis *et al.* 2010; Lo *et al.* 2012). In addition, during meiosis the conserved checkpoint kinases, Mec1 and Tel1, promote recombination between homologs and control the meiotic recombination checkpoint (Grushcow *et al.* 1999; Carballo *et al.* 2008). Finally, the meiosis-specific kinase, Mek1, is required for promoting recombination between homologous chromosomes instead of sister chromatids as well as the meiotic recombination checkpoint (Xu *et al.* 1997; Wan *et al.* 2004; Niu *et al.* 2005; Kim *et al.* 2010). Understanding the mechanisms by which these kinases control various meiotic processes requires the identification of their substrates, coupled with functional analyses of mutants that are unable to be phosphorylated.

In vegetative yeast cells, combining stable isotope labeling by amino acids in cell culture (SILAC) with phosphopeptide purification and mass spectrometry (MS) has been successful in identifying putative kinase substrates (Zhou *et al.* 2010) . This approach relies on the natural metabolism of cells to incorporate heavy isotopes of specific amino acids into the proteome. The increased mass of the proteins generated using these “heavy” amino acids makes them isotopically distinct by MS from proteins from control cells (Ong *et al.* 2002). Therefore, a requirement for SILAC is the ability to grow in synthetic medium so that the presence of either heavy or light amino acids can be controlled. The inefficiency with which *S. cerevisiae* cells sporulate after pre-growth in standard synthetic medium has precluded the application of SILAC to meiotic cells. We have now developed a synthetic pre-sporulation medium that supports efficient sporulation of *S*. *cerevisiae* SK1 diploids, thereby making SILAC experiments in meiotic cells possible. Importantly, analysis of various meiotic landmarks indicates that pre-growth in this synthetic medium results in delayed, but otherwise normal, meiosis.

As proof of principle, our SILAC protocol tested to see if it could identify amino acids on proteins known to be phosphorylated by Mek1, modeled on an approach previously developed for identification of Cdk substrates in vegetative cells (Holt *et al.* 2009). This strategy involves growing a strain containing an analog-sensitive version of the kinase (*mek1-as*) in the presence of either light or heavy arginine and lysine, arresting the cells, and then adding inhibitor for a short time to the heavy culture to inactivate the kinase. Proteins from each culture are then isolated, combined together, and digested with trypsin to generate peptides. Using immobilized metal affinity chromatography (IMAC), phosphopeptides are purified and the light-to-heavy ratio of specific peptides is determined (Ficarro *et al.* 2002; Chen *et al.* 2010). When phosphates added to proteins by the kinase of interest are removed by phosphatases during the period of kinase inactivation, they cannot be replaced; therefore, these phosphopeptides should be under-represented in the heavy culture, resulting in a ratio of light to heavy phosphopeptides greater than 1. Mek1 is an excellent kinase to use as a test case because its activity is constitutively required to maintain the meiotic prophase arrest conferred by deletion of the meiosis-specific recombinase, *DMC1* (Wan *et al.* 2004; Niu *et al.* 2005), there is a well-characterized analog-sensitive allele of *MEK1*, *mek1-as*, and *in vivo* targets of Mek1 are already known (Mek1 T327, Rad54-T132, and Histone H3 T11), thereby allowing validation of the approach (Niu *et al.* 2007; Niu *et al.* 2009; Govin *et al.* 2010). All three of these substrates match the consensus sequence for Mek1 phosphorylation (RXXT/S) determined by screening peptide libraries (Mok *et al.* 2010).

## Materials and Methods

### Yeast strains

All yeast strains are derived from the efficiently sporulating SK1 background and their genotypes are given in Table 1. The construction of the *dmc1∆ mek1-as lys4∆ arg4* diploid, NH2092, used in the SILAC experiment took several steps. First, *MEK1* was deleted from DKB187, which contains *dmc1∆*::*LEU2*, using the polymerase chain reaction (PCR)-mediated deletion approach with the *kanMX6* cassette (Longtine *et al.* 1998). The *LYS4* gene was then similarly deleted using *hphMX4* (Goldstein and McCusker 1999). All knockouts were confirmed either by PCR or by phenotypic analysis. To introduce the *arg4-Nsp* allele, DKB187 *mek1∆ lys4∆* was crossed to S2683 (Hollingsworth *et al.* 1995) and *MAT***a** and *MATα* segregants containing *dmc1∆*::*LEU2*, *mek1∆*::*kanMX6*, *lys4∆*::*hphMX4* and *arg4-Nsp* were selected. These haploids were transformed with the *mek1-as URA3* plasmid, pJR2 (Callender and Hollingsworth 2010), digested with *Rsr*II to target integration of the plasmid immediately adjacent to *mek1∆*::*kanMX6*. The transformants were then mated to make NH2092.

**Table 1:** *S. cerevisiae* strains

Name	Genotype	Source
NH144	*MATα leu2-K HIS4arg4-Nsp* ura3 *lys2* *ho∆::LYS2*	Hollingsworth *et al.* 1995
	*MAT***a** *leu2*::*hisG his4-X ARG4 ura3 lys2 ho∆*::*LYS2*	
NH716	*MATα leu2*::*hisG his4-X*::*LEU2(NgoMIV)* *ho∆::hisG* *ura3(∆pst-sma)*	Callender and Hollingsworth 2010
	*MAT***a** *leu2*::*hisG HIS4*::*LEU2(Bam) ho∆*::*hisG ura3(∆pst-sma)*	
S2683	*MATα leu2-K arg4-Nsp ura3 lys2 ho∆*::*LYS2*	Hollingsworth *et al.* 1995
DKB187	*MAT***a** *leu2*::*hisG his4-X ura3 lys2 ho*::*LYS2 dmc1∆*::*LEU2*	Doug Bishop
NH2092	*MATαleu2 arg4-Nsp ho∆*::*LYS2lys2ura3lys4∆*::*hphMX4*	This work
	*MAT***a** *leu2 arg4-Nsp ho∆*::*LYS2 lys2 ura3 lys4∆*::*hphMX4*	
	*dmc1∆*::*LEU2mek1∆*::*kanMX6*::*URA3*::*mek1-as*	
	*dmc1∆*::*LEU2 mek1∆*::*kanMX6*::*URA3*::*mek1-as*	

### Media

YPD, YPA, and Spo medium are described elsewhere (Lo and Hollingsworth 2011). To make RPS medium, a drop-out powder lacking arginine and lysine is first created by combining 5.0 g each adenine-HCl (Sigma #A8751), uracil (Sigma #U0750), tryptophan (Sigma #T0254), histidine-HCl (Sigma #H8125), and methionine (Sigma #M9625), 7.5 g each tyrosine (Sigma #T3754), leucine (Sigma #L8000), isoleucine (Sigma #I2752), valine (Sigma #V0500), threonine (Sigma #T8625), and serine (Sigma #S4500), and 12.0 g phenylalanine (Sigma #P2126) in a blender. The nutrients are mixed together by 5× 1-min pulses and the resulting −Arg −Lys powder is transferred to a sterile bottle. To make a 250× solution of light lysine and arginine (3% lysine, 2% arginine), 3 g L-lysine-HCl (Sigma #L5626) and 2 g l-arginine-HCl (Sigma #A5131) are dissolved in 100 ml water and the solution is filter-sterilized. The heavy arginine and lysine amino acids contain stable heavy isotopes of both ^13^C and ^15^N. To make a 250× heavy lysine and arginine stock, 0.3 g L-lysine:2HCl (Cambridge Isotope #CNLM-291-H) and 0.2 g l-arginine:HCl (Cambridge isotope # CNLM-539-H) are resuspended in 10 ml water and filter-sterilized. Both light and heavy amino acid stocks are stored in the dark at room temperature. The heavy amino acids are expensive and more stable in powder form; therefore, smaller stocks are made and used within 2 months.

RPS medium is made by dissolving 28 g yeast nitrogen base without amino acids, 20 g potassium acetate, and 8 g −Arg −Lys powder in a total volume of 1 L in a 2-liter beaker on a stir plate at room temperature. After filter sterilization using a Stericup 0.22 μM filter apparatus (Millipore), 12.5 ml 40% sterile glucose is added to a final concentration of 0.5%. RPS-L or RPS-H is created by the addition of 1.6 ml 250× light or heavy Lysine/Arginine solution, respectively, to 400 ml RPS medium. RPS medium should be stored in the dark at room temperature. Fresh RPS medium exhibits a light green color. After 3 days, the color begins to get darker, and this correlates with less efficient sporulation. Therefore, RPS medium should be used within 3 days.

### Sporulation conditions

Sporulation of cells after YPA pregrowth is described elsewhere (Lo and Hollingsworth 2011). The following protocol describes sporulation of cells after pre-growth in either RPS-L or RPS-H. On the first day, a single colony is used to inoculate 2 ml YPD, which is then grown at 30° on a roller between 14 and 24 hr. Part of the colony is patched onto YP glycerol plates to ensure that the colony is not petite (Lo and Hollingsworth 2011). The maximum volume of Spo medium for which efficient sporulation has been observed after RPS pre-growth is 200 ml. To ultimately obtain this volume of sporulating culture, overnight cultures are diluted the next day 1:2000 in RPS-L or RPS-H medium in a 2-liter flask (200 μl into 400 ml RPS-L or H). Aeration is important for both pre-growth in RPS and sporulation, so there should always be a flask volume:culture ratio of at least 5:1. The culture is incubated at 30° in a shaker at 250 rpm until it reaches an optical density (OD_660_) of 1.4 to 1.8. Depending on the strain, this can take from 24 to 48 hr. The cells are pelleted by centrifugation and washed once with sterile water. The cells are then resuspended at a cell density of 3 × 10^7^ cells/ml in a 2-liter flask [for a conversion chart of OD to cell density, see (Lo and Hollingsworth 2011)]. The culture is then incubated in a 30° shaker to allow sporulation. Sporulation efficiency can be monitored by phase contrast microscopy to determine the fraction of cells that form spores. Sporulation efficiency for wild-type strains after pre-growth in either RPS-L or RPS-H should be ∼80%.

### Timecourse analysis

The method for using flow cytometry to monitor premeiotic DNA replication is described in (Wan *et al.* 2006). DSB repair and crossover formation were analyzed using the *HIS4/LEU2* hotspot as described previously (Hunter and Kleckner 2001). This hotspot contains a double strand break (DSB) site flanked by *Xho*I restriction sites. Diagnostic parental and recombinant *Xho*I restriction fragments are visualized by Southern blot analysis. DNA was isolated using the Epicentre Yeast DNA extraction kit from Illumina. DNA was digested with *Xho*I and probed with the 0.6 kb AgeI/*Bgl*II fragment from pNH90 (from Neil Hunter, University of California, Davis). DSBs and crossovers were quantitated using MultiGauge software with a Fujifilm FLA-7000 phosphoimager. To monitor meiotic progression, nuclei were stained with 4′,6-diamindino-2-phenylindole (DAPI) and examined by fluorescence microscopy.

### SILAC labeling of proteins from a *dmc1*Δ *mek1-as* diploid in meiosis

The *dmc1∆ mek1-as* diploid, NH2092, was pre-grown in either RPS-L or RPS-H (hereafter referred to as the “light” and “heavy” cultures, respectively) and transferred to Spo medium; 200 ml cells were incubated in a 2-liter flask in a 30° shaker for 10 hr to allow the cells to arrest with unrepaired DSBs. At this time, 21 μl of dimethyl sulfoxide (DMSO) was added to the light culture and 21 μl 10 mM 1-NA-PP1 [4-amino-1-*tert*-butyl-3-(1′napthyl)pyrazolo[3,4-*d*]pyrimidine] (Tocris Bioscience) dissolved in DMSO was added to the heavy culture (1 μM final concentration). After 20 min, 7 ml cells from each culture were transferred to a 25-ml flask and returned to the 30° shaker until the next day. Inhibition of Mek1-as allows DSB repair by Rad51, thereby eliminating the signal to the checkpoint and allowing the cells to sporulate (Wan *et al.* 2004). Therefore, the effectiveness of both the *dmc1∆* arrest and the Mek1-as inhibition can be determined by analyzing the percent sporulation of the light and heavy cultures. The remaining 193 ml of cells from light and heavy sporulating cultures were then collected by centrifugation and washed once with 40 ml cold sterile water. After the cells were resuspended in cold sterile water, they were transferred to 50-ml conical tubes and the cells were collected again by centrifugation. After pouring off the supernatants, the cell pellets were stored at −80°.

### Preparation of crude chromatin and trypsin digestion

All the quantities described in this protocol are meant for 1 ml yeast pellet volume, unless otherwise specified. To enrich for chromosome-associated proteins, crude chromatin is prepared. The heavy and light cell frozen pellets are thawed by adding 10 mL of 30° reducing buffer [100 mM Tris, pH 9.4, and 10 mM dithiothreitol (DTT) (DTT is added fresh from a 1-M freezer stock)] and incubating at 30° for 15 min. The cells are pelleted by centrifugation at 3700×*g* for 5 min at room temperature. The temperature of the centrifuge is then lowered to 4° so that it is ready for the next spin. The volume of the cell pellet is measured and, for every milliliter of cell pellet, 10 ml of 30° spheroplasting solution (50 mM KPO_4_, pH 7.5, 1.0 M Sorbitol and 10 mM DTT) are added. The cell walls are removed to create spheroplasts by adding 100 μl of 25 U/μL Lyticase (Sigma), and then incubating at 30° for 45 min with rotation. To determine the efficiency of spheroplasting, 20 μL of cells are added to 1 mL of 0.1% sodium-dodecyl-sulfate (SDS) and the cells are examined by phase contrast microscopy for lysis. Lysed cells look gray, whereas unlysed cells remain bright and refractile. After >80% spheroplasting is achieved (usually ∼45 min), the spheroplasts are pelleted at 1500×*g* for 5 min at 4° and washed with 10 mL of ice-cold washing buffer (0.1 M KCl, 50 mM HEPES, pH 7.5, 1.0 M Sorbitol). There is no need to resuspend the pellet during this wash; instead, dislodge the pellet from the bottom of the tube by lightly blowing the edges of the pellet with the wash buffer using a P1000 pipette. After harvesting the cells as before, resuspend the pellet in 1 ml of EB buffer [0.1 M KCl, 50 mM HEPES, pH 7.5, 5 mM EDTA, 50 mM NaF, 5 mM β-glycerol phosphate, 1 mM PMSF, and 2× complete, Mini EDTA-free protease inhibitor cocktail (Roche, made fresh every time)] gently with a 1-ml pipette. To lyse the cells, 50 μl 10% Triton X-100 is added (0.5% final concentration) and the spheroplasts are incubated at 4° with gentle rotation for 15 min. This step can be performed without rotation if the suspension is too thick and mixed periodically with a pipette instead. The lysate is then gently placed on top of a one-sample volume of a 30% sucrose cushion in an ultracentrifuge tube (Beckman #326819) and centrifuged at 30,000×*g* (SW50 or SW55Ti; Beckman Ultracentrifuge) for 15 min at 4°. While loading the sample, it is important to make sure that the boundary between the sample and the sucrose cushion is completely planar. Any unevenness can lead to the sample breaking through the cushion, leading to failure of fractionation. After centrifugation, the supernatant is checked for a yellow tinge. An absence of yellow tinge suggests a failure to fractionate and that the sample should be discarded. The supernatant is removed and the chromatin pellet is thoroughly resuspended in 2 mL Urea Extraction Buffer (8 M Urea, 100 mM Tris pH 8.0, 300 mM KCl, and 10 mM DTT) and incubated at 37° for 30 min with rotation. The resuspended pellet is then centrifuged as before, but for 15 min at 20°. The supernatant is transferred to a new 50-ml conical tube. The pellet is extracted once more with 1 mL of Urea Extraction Buffer and the supernatants combined. At this point, the pellet should appear almost white due to all proteins being extracted from it. The protein concentration of each chromatin preparation is measured using the BioRad Protein Assay reagent. Equal amounts (ideally 10 mg) of light and heavy crude chromatin preparations are combined in a 50 conical tube; 500 mM Iodoacetamide is added to the crude chromatin to a final concentration of 30 mM and incubated for 10 min at room temperature in the dark. Iodoacetamide alkylates the reduced cysteine residues, thereby preventing the reformation of disulfide bonds. The chromatin preparations are then diluted five-fold with TBS (50 mM Tris-HCl, pH 8.0, 150 mM NaCl) so that the concentration of urea is less than 2 M. To digest the proteins into peptides, trypsin [30 mg TPCK-treated trypsin (Worthington, TRTPCK) in 0.1% acetic acid] is added so that the amount of trypsin is equal to 1/100th the amount of chromatin protein. The chromatin is incubated with the trypsin with rotation for 15 hr at 37°. The resulting peptides are acidified by addition of 10% tri-fluoroacetic acid (TFA) to a final concentration of 0.2% and then centrifuged at 3700×*g* to remove insoluble material. During this time, C18 columns are prepared by washing sequentially with 1 column volume (column volume is indicated on the side-wall of the column) of methanol, 80% acetonitrile/0.1% acetic acid, 0/1% acetic acid, and finally 0.1% TFA. The peptides are de-salted by loading the supernatant onto a 500 mg Sep-Pak C_18_ column (Waters) at a ratio of 10 mg total protein per 1 g of C18 resin, washed with 1 column volume of 0.1% TFA using gravity flow, followed by two column volumes of 0.1% acetic acid. Peptides are eluted using 3 ml 80% acetonitrile, 0.1% acetic acid. To maximize the yield, collect any liquid remaining in the column by placing a rubber bulb on top of the column and pushing the liquid out. The peptide solution is aliquoted into glass cuvettes (National Scientific, C4015-843) and dried under vacuum until the volume is reduced sufficiently to fit into one cuvette (approximately 1 hr). The walls of the cuvettes are washed with 80% acetonitrile, 0.1% acetic acid, and then everything is pooled into one cuvette. This cuvette is then placed back into the speedvac overnight to completely dry the peptides.

To measure the efficiency of heavy amino acid labeling, 1 mg of heavy crude chromatin alone is treated with iodoacetamide and trypsin as described above and analyzed using the mass spectrometer.

### Preparation of columns for immobilized metal affinity chromatography (IMAC)

Gel loading tips (Fisher #02-707-138) are used to make IMAC columns. A small bunch of glass fibers (Fisher #11-388) is carefully placed inside the top half of the tip and is then carefully maneuvered with a piece of thin wire into the narrow part of the tip. Glass fibers are continuously pushed through the tip until they will not move any further. At this point, excess fibers protruding from the tip are cut off with scissors, flush with the end of the tip. The end of the tip containing the frit is bent and flattened with the back of a forceps to decrease the flow rate. The top part of the tip is trimmed until the diameter is small enough so that a 1-ml syringe can fit snugly, creating an airtight seal. To test the column, 10 µl water is loaded into the tip from the top, and the syringe is attached to the tip with the plunger drawn up all the way. If water flows out when pushed with the syringe, then the column tip is ready to use.

Resin from three silica-nitrilotriacetic acid spin columns (Qiagen, Valencia, CA) is pooled in 50 ml of stripping buffer (5 mM EDTA, pH 8.0, 1 M NaCl) in a 50-ml conical tube and incubated for 1 hr at room temperature with rotation. To collect the resin from the columns, each column is turned upside-down over the 50-ml tube and the frit and dry resin are carefully pushed out using a blunt needle. Gently tap the column to collect resin that is stuck to the column wall. The resin is pelleted by centrifugation at 1500×*g* and washed with 50 ml water, followed by a 50-mL wash of 0.6% acetic acid. The resin is then incubated with 100 mM FeCl_3_ in 0.3% acetic acid overnight at room temperature with rotation. The resin is washed once with 50 ml 0.6% acetic acid, and then twice with 50 ml 0.1% acetic acid. The amount of resin is estimated by comparison with the volume of water in a comparable tube so that the resin can be suspended in 0.1% acetic acid as a 50% (volume/volume) slurry and stored at 4°. The final product should have a yellow tinge.

The appropriate amount of packed resin required for IMAC is calculated (40 μl packed resin/5 mg peptides) and the same amount of water is loaded into the top of the column tip. A 1-ml syringe is attached to the tip and water is pushed very slowly until its lower level touches the glass frit. The syringe is removed immediately to prevent the water from flowing through. The upper level of the water is marked, and then it is forced out with the syringe. The 50% resin slurry is loaded into the tip and slowly pushed with the syringe to pack the resin until the water meniscus touches the resin bed. Once the packed resin reaches the mark for the correct volume, the column is ready. Push out all the excess liquid, but do not allow the resin to dry.

### Purification of phosphopeptides using IMAC

The method for purifying phosphopeptides using IMAC resin is adapted from Albuquerque *et al.* (2008). The dried peptides are resuspended in 100 μl 0.1% acetic acid (10 μg/μl peptide concentration) and spun for 5 min at 2300×*g* in a microcentrifuge. The supernatant is loaded using a micropipette onto a gel loading tip column with fiber glass as a frit containing the appropriate amount of fresh immobilized metal affinity chromatography (IMAC) resin (usually ∼60 μl; see above). After loading, the IMAC resin is washed twice with 60 μL of wash buffer containing 25% acetonitrile, 100 mM NaCl, and 0.1% acetic acid, once with 60 μL 0.1% acetic acid, and finally with 30 μl water. Loading and washes are performed by slowly pushing the buffer through the column using a syringe at a flow rate of 1 to 2 μl/min. The syringe is attached to the tip with the plunger fully drawn, and then the plunger is pushed from the 1 ml to the 0.9 ml marking. This causes the sample to slowly ooze out of the tip. The flow-through is collected in a glass cuvette that can be freeze-dried and used later in case the first round of IMAC fails to purify any phosphopeptides. The column is allowed to run with close monitoring to make sure that the meniscus of the liquid does not drop below the bed level. Once the meniscus reaches the bed, the syringe is removed and next wash solution is added to the top of the column. The plunger is drawn to 1 ml and the syringe is reattached and used as before. The resin should not be allowed to dry at any point (the meniscus of each loading or wash should be right at the resin surface). Phosphopeptides are eluted into a new glass cuvette using 180 μl 6% NH_4_OH and then dried under vacuum. The dried phosphopeptides can be stored at −20° until ready for MS.

### HILIC (hydrophilic interaction liquid chromatography) fractionation and mass spectrometry

Phosphopeptides eluted from IMAC were subjected to an offline HILIC fractionation method as previously described (Albuquerque *et al.* 2008). Fractionated samples were run on an LTQ Orbitrap XL using a 1100 Quad PUMP HPLC system (Agilent, Santa Clara, CA) with Ultimate 3000 autosampler (Dionex, Sunnyvale, CA).

### Data analysis using SEQUEST

To search tandem mass spectra, a composite database is used containing both the yeast protein database and its decoy database. The use of a reverse protein database allows for the estimation of the false discovery rate (Elias and Gygi 2007). The data are analyzed using the Sorcerer system (SageN, San Jose, CA; SEQUEST) and a semi-tryptic restriction is applied to the search. The parameters used for the search are a parent mass tolerance of 20 ppm, +80.0 Da variable modification of STY (serine, threonine, and tyrosine) due to phosphorylation, and a maximum of two modifications per peptide. After searching, the raw results of SEQEST are then filtered using their provided *p* value to a 1% false discovery rate (number of decoy database hits/total hits).

## Results

### Pre-growth in RPS-L medium allows for normal meiosis and sporulation

Synthetic dextrose (SD) medium for growth of budding yeast cells usually comprises 0.67% yeast nitrogen base without amino acids and 2% glucose, which can then be supplemented with appropriate amino acids and nucleotides to final concentrations of between 20 and 400 mg/L (Rose *et al.* 1990). Cells pregrown in SD medium plus nutrients do not sporulate efficiently when transferred to Spo medium (data not shown). Sporulation in SK1 strains is enhanced by pre-growth in a rich acetate medium called YPA (Padmore *et al.* 1991). To create a synthetic sporulation medium (RPS) that supports efficient sporulation, the major carbon source in SD was changed from glucose to 2% acetate, similar to YPA. A small amount of glucose (0.5%) is included and is necessary for cells to grow well. In addition, four-times the amount of dropout powder is used compared with conventional SD dropout medium (see *Materials and Methods* for recipes). “Heavy” versions of lysine and arginine contain stable heavy isotopes of ^13^C and ^15^N. RPS supplemented with “light” or “heavy” arginine/lysine is indicated as RPS-L and RPS-H, respectively.

**Figure 1:**
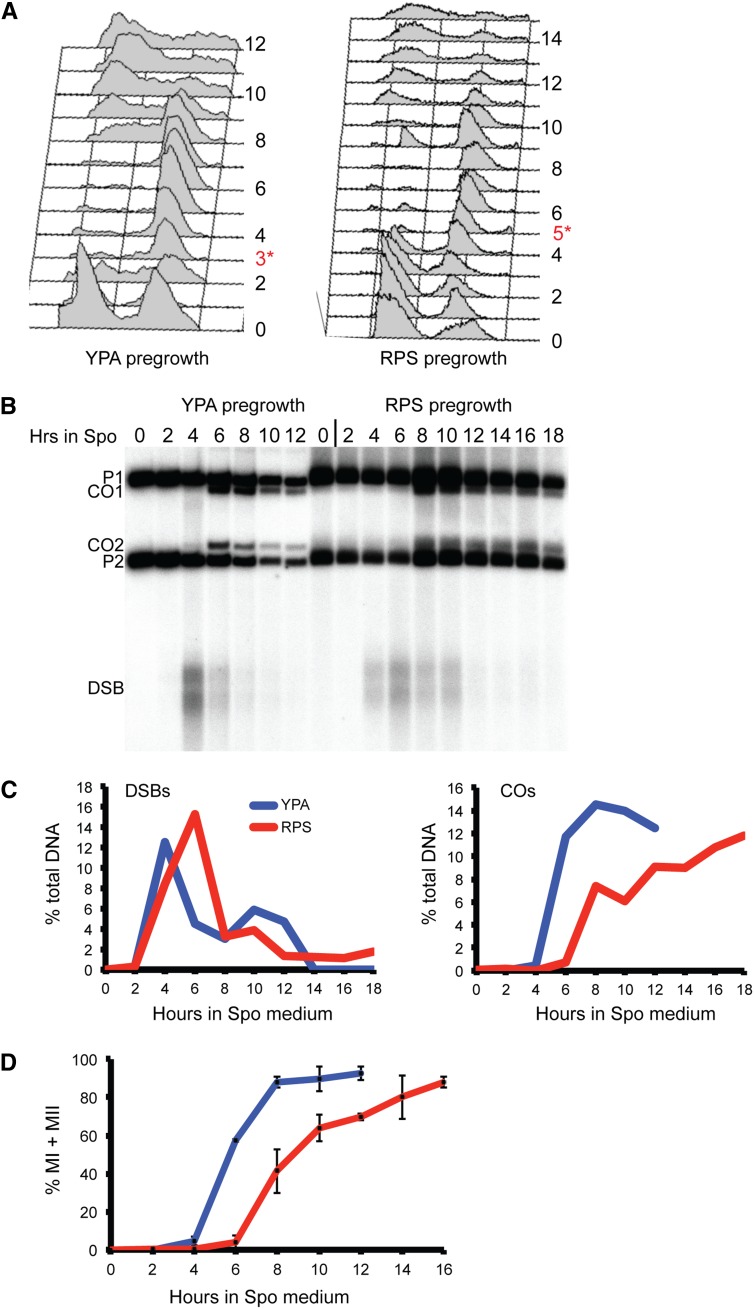
Comparison of various meiotic parameters in cultures grown in either YPA or RPS-L prior to sporulation. The wild-type SK1 strain, NH716, was pre-grown in either YPA or RPS-L and transferred to Spo medium at 30°, and samples were taken at 2-hr intervals. (A) Flow cytometry analysis of premeiotic DNA synthesis. Numbers indicate hours after transfer to Spo medium. Red numbers with asterisks indicate the time points at which DNA synthesis is complete. (B) DSB and crossover analysis was performed using the *HIS4/LEU2* hotspot (Hunter and Kleckner 2001). P1 and P2 indicate parental fragments, CO1 and CO2 indicate crossover fragments, and DSB indicates DSB fragments. This analysis was performed for two different time courses with similar results. (C) Quantitation of the DSBs and COs shown in (B). (D) Meiotic progression was measured by staining cells with DAPI and counting the number of nuclei in each cell (binucleates indicate completion of Meiosis I and tetranucleates indicate completion of Meiosis II). For each time point, 200 cells were examined by fluorescent microscopy. Error bars represent the SD observed for the two independent experiments.

Although a slight reduction in sporulation (84% from 95%) was observed in two different wild-type strains pre-grown in RPS-L compared with YPA, spore viability was wild-type (Table 2). Timecourse analyses were used to monitor various meiotic parameters. Cells pre-grown in RPS-L exhibit a 2-hr delay in the onset of premeiotic DNA synthesis compared with YPA pre-growth (Figure 1A). Once the cells enter meiosis, however, meiotic parameters such as DSB and crossover formation, as well as meiotic progression, occur with normal timing (taking into account the initial delay) (Figure 1, B–D). These results indicate that the transition from vegetative growth to meiosis takes longer after pre-growth in RPS medium but, having once entered meiosis, cells proceed normally and relatively synchronously to form highly viable spores.

**Table 2:** Sporulation and spore viability under various conditions after pre-growth in YPA, RPS-L, or RPS-H

Strain	Relevant Genotype	Pregrowth Medium	% Sporulation*^a^*	% Spore Viability*^b^*
NH144	WT	YPA	94.5 ± 1.4	96.3 ± 1.8
		RPS-L	83.4 ± 4.6	95.6 ± 0.9
NH716	WT	YPA	95.5 ± 0.7	95.0 ± 1.8
		RPS-L	84.3 ± 1.1	95.0 ± 3.5
NH2092	*mek1-as dmc1∆* - I	YPA	0.0 ± 0.0	ND
	*mek1-as dmc1∆* - I	RPS-L	0.0 ± 0.0	ND
	*mek1-as dmc1∆* - I	RPS-H	0.0 ± 0.0	ND
	*mek1-as dmc1∆* +I*^c^*	YPA	91.0 ± 2.1	ND
	*mek1-as dmc1∆* +I	RPS-L	80.1 ± 1.8	ND
	*mek1-as dmc1∆* +I	RPS-H	84.0 ± 2.8	ND

a Numbers represent the average and SD from two independent experiments.

b Spore viability was determined by dissection of 20 tetrads for each experiment.

c "I" indicates addition of 1-NA-PP1 to a final concentration of 1 μM. Inhibitor was added to the YPA and RPS pregrown strains 5 hr and 10 hr after transfer to Spo medium, respectively.

### Pre-growth in RPS-H efficiently labels proteins both in vegetative cells and after the induction of meiosis

For SILAC experiments to work, the labeling of proteins with heavy amino acids must be highly efficient. Peptides from RPS-H grown vegetative cultures were analyzed by MS, and 3900 out of 4064 total identified peptides (96%) contained heavy amino acids. One question is whether the fraction of heavy labeled proteins remains high after hours in Spo medium. Meiosis and sporulation are induced by transferring cells into a medium that lacks nitrogen and contains a nonfermentable carbon source such as acetate. The absence of nitrogen means that no new amino acid synthesis can occur. Instead, amino acids used for new protein synthesis are recycled from existing proteins by autophagy, as evidenced by the fact that protease activity is essential for sporulation (Zubenko *et al.* 1979; Onodera and Ohsumi 2005). This recycling is an advantage for meiotic SILAC experiments, given that proteins produced during meiosis must be made using heavy amino acids that were present during the pre-growth period. Consistent with this idea, 5443 out of 5611 (97%) of the identified peptides analyzed 10 hr after transfer to Spo medium from RPS-H pre-grown cells still contained heavy amino acids.

Another possible problem is that catabolism of heavy arginine could produce proline and glutamic acid residues with heavy atoms that could skew the quantification of heavy peptides (Middelhoven 1964). To determine how frequently this happens, the raw data were analyzed for the presence of heavy proline or glutamate and low levels (<1.0%) of these amino acids were observed. Analysis of the most abundant proline and glutamate-containing peptides exhibited L/H ratios not significantly different than 1. Therefore, catabolism of arginine during meiosis is unlikely to significantly affect the quantification of proline/glutamate-containing peptides.

### SILAC, combined with phosphopeptide purification, identifies known substrates of Mek1

**Figure 2:**
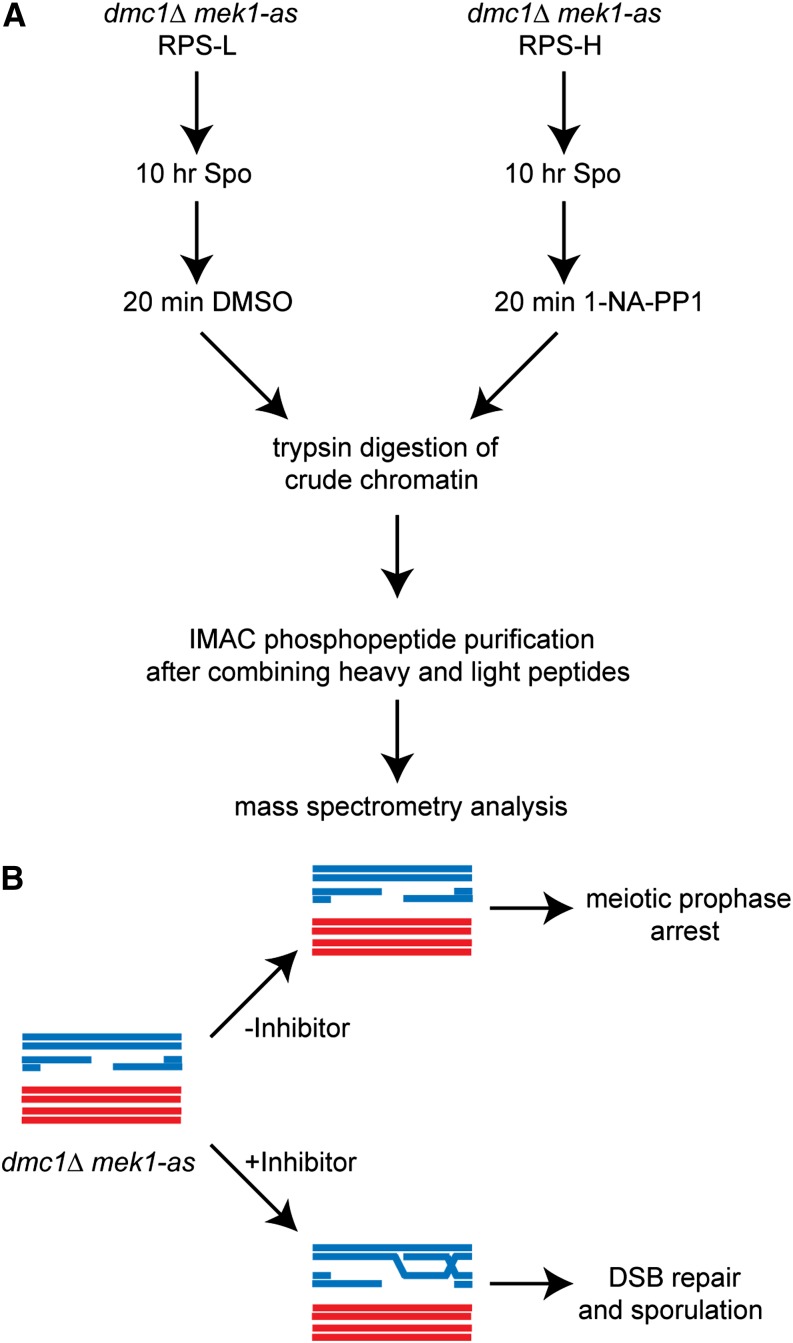
Fate of meiotic DSBs in *dmc1∆ mek1-as* diploids with or without inhibitor. (A) Experimental flow chart for a SILAC experiment using *dmc1∆ mek1-as*. (B) Inactivation of Mek1-as allows DSB repair in a *dmc1∆* mutant. Two homologs (one red and one blue) are shown after DNA replication to make sister chromatids. Each chromatid is represented as a duplex of DNA. DSBs formed on one of the four chromatids remain unrepaired in the absence of the 1-NA-PP1 inhibitor, thereby triggering the meiotic recombination checkpoint and arresting the cells in meiotic prophase (Bishop *et al.* 1992; Lydall *et al.* 1996). Addition of 1-NA-PP1 inactivates Mek1, thereby allowing Rad51 to repair the DSBs using sister chromatids as templates. This repair eliminates the signal to the checkpoint, thereby allowing meiosis to proceed and asci to form (Wan *et al.* 2004; Niu *et al.* 2005).

**Figure 3:**
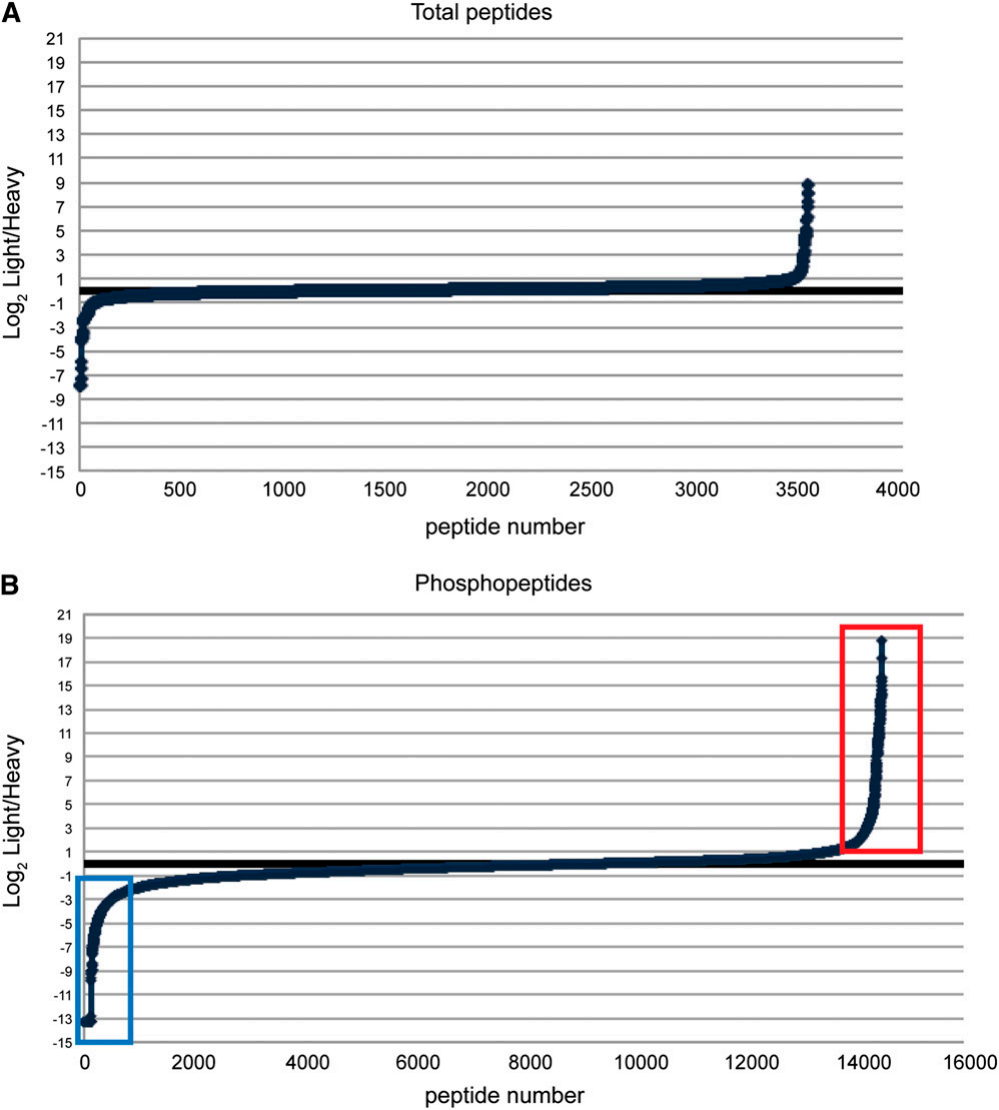
Relative abundance of total and phosphorylated peptides from a *dmc1∆ mek1-as* SILAC experiment. Representative distributions of log_2_ L/H ratios for total peptides are plotted on the Y-axis, whereas an arbitrary peptide identification number is assigned to each individual peptide, shown on the X-axis. Peptides with L/H ratios greater than two-fold are indicated by the red box, whereas peptides with L/H ratios <1 are indicated by the blue box.

To validate the method, a meiotic SILAC experiment was performed to see if it could identify known substrates of Mek1. The experimental strategy used for the *dmc1∆ mek1-as* SILAC experiments is shown in Figure 2A. In the absence of *DMC1*, cells arrest in prophase with resected double strand breaks (Bishop *et al.* 1992) (Figure 2B). Rad51 is loaded onto the breaks, but its activity is indirectly inhibited by Mek1 (Bishop 1994; Niu *et al.* 2009). Inactivation of Mek1-as in *dmc1∆ mek1-as* arrested cells allows repair of the breaks using sister chromatids as templates, thereby removing the signal to the meiotic recombination checkpoint and allowing meiotic progression (Niu *et al.* 2005) (Figure 2B). An overnight culture of a *dmc1∆ mek1-as* diploid was diluted into either RPS-L or RPS-H, the cells were grown to the appropriate density, and then those cells were transferred to Spo medium for 10 hr to allow them to arrest in meiotic prophase with unrepaired DSBs. The effectiveness of both the arrest and Mek1 inhibition were assessed by removing small aliquots of cells before and after addition of the Mek1-as inhibitor, 1-NA-PP1, and incubating them at 30° overnight. Although no asci were observed in the absence of inhibitor, indicating a robust arrest, inactivation of Mek1 resulted in high levels of sporulation as expected (Table 2). For the SILAC experiment, cells were harvested 20 min after addition of inhibitor. Because all known substrates of Mek1 are associated with DNA, crude chromatin was isolated to enrich for proteins that are potential targets of the kinase. The chromatin preparations from the heavy and light cultures were combined and treated with trypsin. Trypsin cuts after arginine or lysine to generate peptides. Analysis of a fraction of the total peptides by MS showed that 96% of the total peptides exhibited an L/H ratio of approximately 1, indicating that most proteins were present in equal abundance in the two chromatin preparations (Figure 3A). Phosphopeptides were then enriched using IMAC. The peptides were subjected to HILIC fractionation prior to MS analysis, which allows for in-depth mapping of protein phosphorylation sites in a complex sample. HILIC is largely orthogonal to reverse-phase high-pressure liquid chromatography for phosphopeptide separation but does not require a de-salting step before LC-MS/MS analysis, preventing sample loss after fractionation (Albuquerque *et al.* 2008). In two different experiments, 16,036 and 16,555 total peptides were identified, of which 15,241 (95%) and 13,959 (84%) were phosphorylated, respectively. The peptides from these two experiments represent 1985 and 2058 unique phosphorylated proteins. Because the purpose of this article is meant to report the method for performing SILAC in meiosis, the entire dataset of identified phosphopeptides will be made available elsewhere.

**Figure 4:**
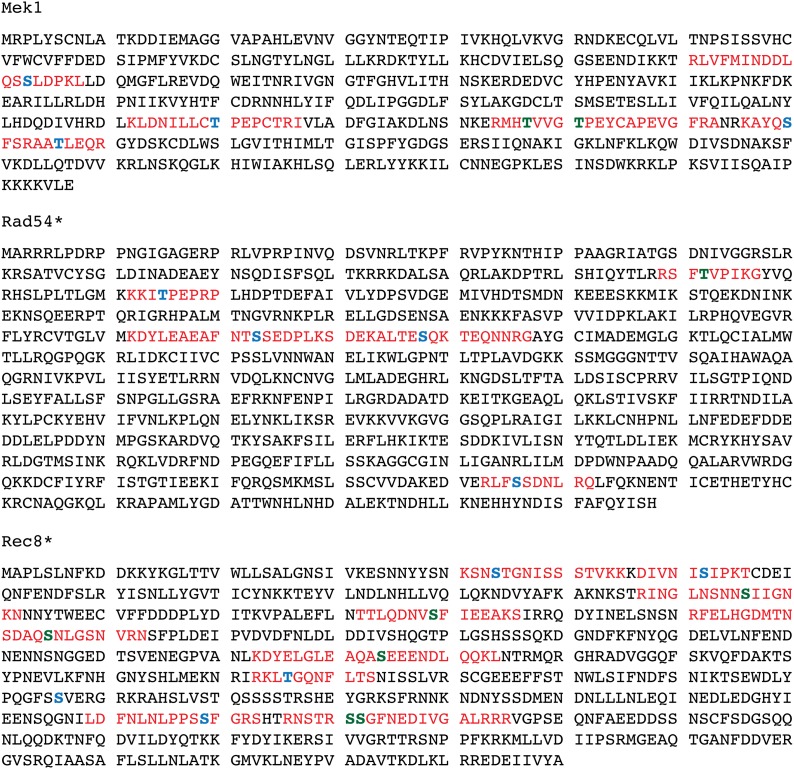
Location of phosphopeptides on Mek1, Rad54, and Rec8. Sequences in red indicate peptides that were detected by mass spectrometry. In some cases, two peptides overlap. Amino acids indicated in bold with blue color are phosphorylated. Phosphorylated sites that have previously been identified in the literature are bold and green.

**Figure 5:**
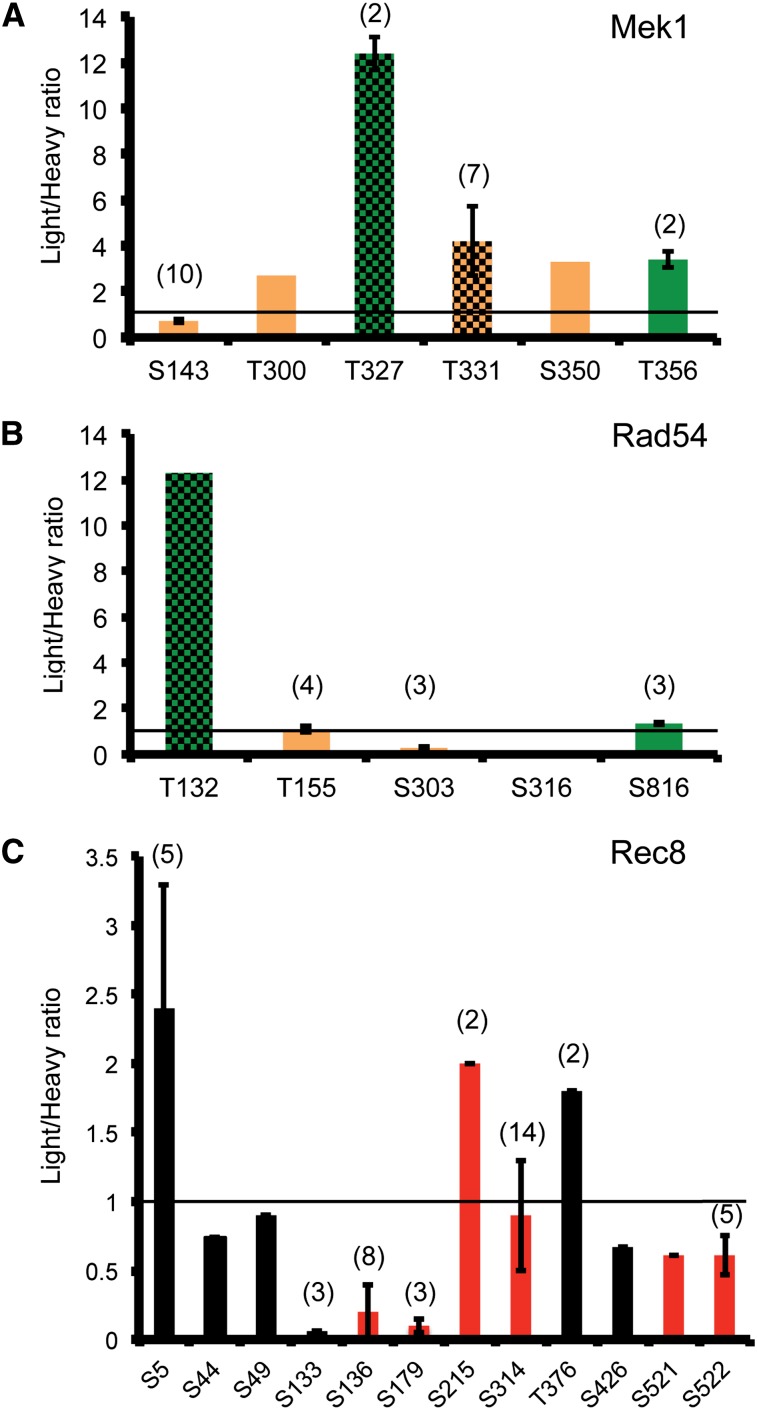
Distribution of phosphorylated peptides and their light:heavy (L/H) ratios for Mek1, Rad54, and Rec8. The average L/H ratios are plotted for peptides from *dmc1∆ mek1-as* SILAC experiments containing the phosphorylated amino acid indicated on the X-axis. Error bars indicate the SD. A complete list of each peptide and its ratio for the proteins shown here can be found in Supporting Information, Table S1. For those phosphosites identified by more than one peptide, the number of peptides is indicated in parentheses. Green indicates phosphosites that match the Mek1 consensus of arginine in the −3 position. Hatch marks indicate *in vivo* Mek1 targets important either for Mek1 auto-activation (T327, T331) or for inhibiting Rad51–Rad54 interaction (T132). For Rec8, red indicates phosphosites previously identified (Brar *et al.* 2006; Katis *et al.* 2010). Phosphorylation of these sites promotes separase cleavage (Katis *et al.* 2010). Lines across each graph indicate a ratio of 1.0.

A plot of the L/H ratios of the phosphorylated peptides shows that approximately 80% of these peptides are equally abundant in the light and heavy cultures (Figure 3B). Peptides that exhibit a L/H ratio >2 (indicated by the red box) are potential substrates of Mek1. The data were examined for phosphopeptides from the three known *in vivo* targets of Mek1: Mek1 threonine 327, Rad54 threonine 132, and histone H3 threonine 11. No peptides for H3 were observed, perhaps because the abundance of arginine and lysine residues in this histone produces very small peptides that are not efficiently detected by MS. In contrast, several phosphopeptides were found for both Mek1 and Rad54 (Figure 4). Mek1 activates itself by autophosphorylation of T327 (Niu *et al.* 2007). Two peptides containing this phosphosite were identified exhibiting an average L/H ratio of 12.4 (Figure 5A). In addition, seven peptides were observed that contain phosphorylated T331, which is also located in the Mek1 activation loop and whose phosphorylation is required for wild-type levels of Mek1 activity, exhibiting an average L/H ratio of 4.2 (Niu *et al.* 2007). High L/H ratios could occur simply because there is less Mek1 protein in the heavy culture. This potential artifact is ruled out in the case of Mek1 because other phosphopeptides (presumably phosphorylated by other kinases) are present that have L/H ratios ≤1. For example, peptides containing phosphorylated serine 143 are present with an average L/H ratio of 0.72 (Figure 4 and Figure 5A), supporting the idea that the large L/H ratios for T327 and T331 are Mek1-dependent.

Another *in vivo* target of Mek1 is threonine 132 of Rad54 (Niu *et al.* 2009). Rad54 is an accessory factor for Rad51 and the negative charge conferred by phosphorylation makes Rad51–Rad54 complex formation more difficult. A high L/H ratio for a peptide containing phosphorylated T132 of Rad54 was observed, consistent with being a Mek1 target (Figure 5B). This ratio is not due to differential amounts of Rad54 in the two cultures as other phosphopeptides from Rad54 exhibited L/H ratios ranging from 0.06 to 1.35 (Figure 4 and Figure 5B).

In addition to peptides exhibiting L/H ratios greater than 1, there is also a class of peptides in which the L/H ratio is less than 1 (represented by the blue box in Figure 3B). These are phosphopeptides that are over-represented in the heavy culture, suggesting that phosphorylation occurs in response to Mek1 inhibition. In fact, inactivation of Mek1 in *dmc1∆*-arrested cells changes the cellular physiology, as DSB repair can now occur, thereby relieving the signal to the checkpoint and allowing meiotic progression (Wan *et al.* 2004; Niu *et al.* 2005) (Figure 2B). Although the inhibitor was present for only 20 min, the presence of phosphopeptides with ratios <1 suggests that this is enough time for at least some proteins that are phosphorylated only after strand invasion to be modified. An excellent example of this type of regulation was observed with Rec8. Rec8 is a meiosis-specific subunit of cohesin, the multisubunit complex that holds sister chromatids together (Klein *et al.* 1999). The spindle checkpoint monitors the attachment of homologous pairs of sister chromatids to opposite poles of this spindle at Meiosis I (Shonn *et al.* 2000). Once all homologous pairs are properly oriented, separase is activated to cleave Rec8 specifically along chromosome arms, thereby allowing reductional segregation (Buonomo *et al.* 2000). This cleavage requires phosphorylation of Rec8 by several kinases, including Cdc7-Dbf4 and Hrr25 (Katis *et al.* 2010). Our SILAC experiments identified eight phosphosites on Rec8 that are present on peptides that exhibit ratios less than 1 (Figure 5C). Five of these have been previously identified by MS studies of Rec8 enriched from meiotic cells and phosphorylation of these amino acids helps promote separase cleavage (Figure 4) (Brar *et al.* 2006; Katis *et al.* 2010). Therefore, our MS data suggest that phosphorylation of these sites on Rec8 does not occur until after strand invasion.

## Discussion

SILAC requires cells to be grown in medium containing either light or heavy amino acids so that proteins coming from different cultures can be distinguished by the differences in their mass. Controlling the source of amino acids requires the use of synthetic medium. A problem for using SILAC for studying meiosis is that cells pre-grown in standard synthetic medium do not sporulate well. Our development of a synthetic medium that allows efficient sporulation has enabled the application of SILAC to meiotic yeast cells for the first time. In comparison to YPA pre-growth medium, there is an approximate 2-hr delay in the onset of premeiotic DNA synthesis after pre-growth in RPS-L. This delay may be due to the need to deplete particular nutrients such as glucose before entry into meiosis. Importantly, after pre-growth in RPS-L, meiotic recombination and progression occur relatively synchronously and with high efficiency to produce viable spores.

One way to identify kinase substrates using SILAC is to arrest cells containing an analog-sensitive allele of the kinase, and then to inhibit the kinase in either the heavy or the light culture (Holt *et al.* 2009). For meiotic studies, a number of different arrest points are available. In addition to the *dmc1∆* arrest used in this work, *ndt80∆* can be used to arrest cells in pachytene when homologous chromosomes are completely synapsed and double Holliday junction intermediates have formed, and *pCLB2-CDC20* can be used to arrest cells in Metaphase I, after synaptonemal complex disassembly and crossover formation are complete (Xu *et al.* 1995; Allers and Lichten 2001; Lee and Amon 2003). In addition, analog-sensitive kinases have been developed for several kinases known to play key roles in meiosis, including *CDC7*, *CDC28*, *IME2*, and *HRR25* (Benjamin *et al.* 2003; Wan *et al.* 2008; Katis *et al.* 2010). An alternative approach to using analog-sensitive kinase mutants is to induce transcription of a gene encoding a wild-type kinase during an arrest. For example, induction of *CDC5*, the polo-like kinase in budding yeast, in *ndt80∆* arrested cells is sufficient for Holliday junction resolution and synaptonemal complex disassembly (Sourirajan and Lichten 2008). Therefore, by inducing *CDC5* in *ndt80∆* cells specifically in the light culture, a high L:H phosphopeptide ratio would be expected for Cdc5 substrates. SILAC can now be used to look at the function of these kinases (and others) at various times during meiosis.

Our method was validated by showing that known targets of Mek1 can be detected with high L:H ratios from *dmc1∆* arrested cells. One of these peptides contained Mek1 T327, which is present in the Mek1 activation loop and is autophosphorylated *in trans* to activate the kinase (Niu *et al.* 2007). In addition, peptides with L:H ratios >2 were observed for Mek1 T331. These ratios suggest that Mek1 may be the kinase that phosphorylates T331, even though this threonine is not contained within a Mek1 consensus site (Mok *et al.* 2010). It should be noted that the experimental approach of inhibiting the kinase for a brief time during an arrest may underestimate the number of kinase substrates. This is because high L:H ratios are predicted for peptides containing phosphorylation sites based on the assumption that phosphatases can remove phosphates from substrates during the time that the kinase is inactivated, which may not always be true. In addition, the data can also be analyzed to determine proteins with L/H ratios <1, which may represent phosphorylation that is happening as a result of kinase inhibition, as was seen for Rec8.

Although our goal is to define kinase substrates in meiosis, SILAC has other applications as well. For example, SILAC can also be used to look at protein–protein interactions (Blagoev *et al.* 2003). By allowing meiotic proteins to be labeled with heavy amino acids, our protocol opens the door for many different SILAC applications to meiosis.

## Supplementary Material

Supporting Information
