# Supplementary material for: A Method for Sporulating Budding Yeast Cells That Allows for Unbiased Identification of Kinase Substrates Using Stable Isotope Labeling by Amino Acids in Cell Culture
Source: G3 (Bethesda). 2014 Aug 27;4(11):2125–35. doi: 10.1534/g3.114.013888 (PMC4232538; doi:10.1534/g3.114.013888)
Supplement: Supporting Information [file supp_4_11_2125__index.html]

A Method for Sporulating Budding Yeast Cells That Allows for Unbiased Identification of Kinase Substrates Using Stable Isotope Labeling by Amino Acids in Cell Culture — Supporting Information 

# A Method for Sporulating Budding Yeast Cells That Allows for Unbiased Identification of Kinase Substrates Using Stable Isotope Labeling by Amino Acids in Cell Culture

## Supporting Information for Suhandynata *et al.*, 2014

**Files in this Data Supplement:**

- Table S1 - Complete list of the each peptide and its ratio for the proteins shown in Figure 5. (.xlsx, 12 KB)
